# Reply to ‘Sigmoidal Acquisition Curves are Good Indicators of Conformist Transmission’

**DOI:** 10.1038/s41598-018-30382-0

**Published:** 2018-09-18

**Authors:** Alberto Acerbi, Edwin J. C. van Leeuwen, Daniel B. M. Haun, Claudio Tennie

**Affiliations:** 1 Eindhoven University of Technology, School of Innovation Sciences, Eindhoven, 5600 MB The Netherlands; 2 University of St Andrews, School of Psychology & Neuroscience, Westburn Lane, St Andrews, Fife KY16 9JP United Kingdom; 3 Max Planck Institute for Psycholinguistics, Wundtlaan 1, 6525 XD Nijmegen, The Netherlands; 4 University of Leipzig, Department of Early Child Development and Culture and Leipzig Research Center for Early Child Development, Jahnallee 59, Leipzig, 04109 Germany; 5 Department of Early Prehistory and Quaternary Ecology, University of Tübingen, 72074 Tübingen, Germany

## Abstract

In the Smaldino *et al*. study ‘Sigmoidal Acquisition Curves are Good Indicators of Conformist Transmission’, our original findings regarding the conditional validity of using population-level sigmoidal acquisition curves as means to evidence individual-level conformity are contested. We acknowledge the identification of useful nuances, yet conclude that our original findings remain relevant for the study of conformist learning mechanisms.

Replying to: Smaldino, P. E., Aplin, L. M. & Farine, D. R. Sigmoidal Acquisition Curves Are Good Indicators of Conformist Transmission. *Sci. Rep*. 8, 10.1038/s41598-018-30248-5 (2018).

## Introduction

Smaldino, Aplin & Farine^1^ provide a thorough criticism of a series of models where we identified three scenarios producing a sigmoidal relationship between the frequency of a trait in a population and the probability to copy it (henceforth: the sigmoid), without a conformity bias at individual level^2,3^:

1. When there is a preference for one cultural trait over the other (the condition “*Variant Preference*”)^2^.
2. When the (random) choice of demonstrators is limited to a subset of the population (the condition “*Demonstrators subgroup*”)^2^.
3. When copying probability is plotted against the cumulative frequency of behaviours in the population, rather than against the frequency of individuals showing the behaviour at each time step^3^.

## Discussion

Regarding the scenario “*Variant Preference*”, Smaldino *et al*. show that the sigmoid is produced (in part) because (*i*) the outputs of the simulations are averaged across all runs, and (*ii*) the simulations are initialised by randomly populating the individuals with one of the two variants. This is correct, but we do not believe these assumptions are problematic. First, we intended to show that experiments in which results of different runs are pooled together (as in the key empirical study considered by Smaldino *et al*.^4^) might produce the sigmoid in the absence of conformity, when a stable within-run preference for one of the two variants is present. Second, the decision to initialise randomly each individual in the population with one of the two variants was based on several empirical studies in which conformity was tested after many, if not all, individuals had obtained one or the other cultural variant (e.g.^5–8^). In a model not addressed by Smaldino *et al*., we additionally analysed a comparable scenario in which the sigmoid was caused by random initialisation, and we showed that similar results could be obtained when cultural variants were not randomly initialised but were diffused from a small part of the population^3,9^.

Regarding the scenario “*Demonstrators subgroup”*, Smaldino *et al*. provide a successful replication of our model. The fact that, to produce a sigmoid in absence of conformity at individual-level, the total group size and the total number of demonstrators should be relatively small is studied and reported in detail in our original contribution^2^. Note that the scenario in which empiricists study small populations in which a small set of “leaders” may influence group dynamics (i.e., the model parameters under which the sigmoid may emerge in the absence of individual-level conformity) does not seem exceptional (e.g.^10,11^). Our original finding thus means that the sigmoid cannot be taken as evidence for individual-level conformity unless the alternative mechanism of “copy-any-kind-of-subset of the population” (e.g. the dominants or experts) is ruled out. Such a discriminant analysis may not be complicated^1^, but has, hitherto, been absent from many, if not all, conformity studies (e.g.^4,5,11,12^).

Finally, regarding the third scenario, Smaldino *et al*. state that (*i*) conforming to the majority of “instances” and “individuals” is functionally equivalent in all but the most exceptional of cases (based on^4,13^), and (*ii*) the result that plotting instances as opposed to individuals can lead to an artefactual sigmoid is due to sampling from the entire history of events rather than from a subset. The first aspect refers to an open question. Here, we note that one example of converging results cannot resolve the issue^13^, and that, even in the study on which this assertion is based, there is a sub-population of birds in which following the majority of instances would lead conformists to adopt the opposing cultural variant from when they would follow the majority of individuals (see^4^, Figure 1C, control population “C2”: the majority of instances points to option B, whereas the majority of individuals use option A). Regarding the second aspect – sampling from the entire history of events – we acknowledge that applying a limited time-window to the calculation of the observed distribution of traits may be sensible. At the same time, we note that, to our knowledge, there are no conformity studies other than Aplin *et al*.^4^ that apply any time-window for the calculation of observed trait distributions. In other words, our model concerns other conformity studies and Smaldino *et al*.’s results should be taken as a validation of our cautionary statements in this regard. Note also that the time window of ~2,000 observation events that Smaldino *et al*. identify as the threshold after which the sigmoid can be produced in absence of conformity is not that large, given that events in the models represent individual interactions. To put this into perspective, Smaldino *et al*.’s exemplary study in which a time-window (245 seconds) is applied^4^ presents evidence for the sigmoid based on more than 2,000 interactions (i.e., 367 birds observing a mean of 6 interactions, leading to a total number of 367*6 = 2,202 observation events). This means that the sigmoid as reported in Aplin *et al*.^4^ could have emerged in the absence of individual-level conformity.

## Conclusion

Taken together, Smaldino *et al*. provide nuance to our original results without dismissing the conclusion of our studies^2,3^: the sigmoid cannot be taken as evidence for individual-level conformity unless certain conditions are fulfilled. We have shown that these conditions are often violated in conformity research and encourage follow-up work on the equifinality of (sigmoidal) population-level signatures^14,15^, in particular with respect to the nature of inference: population-level patterns may be best used to exclude, instead of evidence, individual-level processes^16,17^.
