# Author Correction: Reply to ‘Sigmoidal Acquisition Curves are Good Indicators of Conformist Transmission’

**DOI:** 10.1038/s41598-019-40288-0

**Published:** 2019-04-17

**Authors:** Alberto Acerbi, Edwin J. C. van Leeuwen, Daniel B. M. Haun, Claudio Tennie

**Affiliations:** 1 Eindhoven University of Technology, School of Innovation Sciences, Eindhoven, 5600 MB The Netherlands; 2 University of St Andrews, School of Psychology & Neuroscience, Westburn Lane, St Andrews, Fife KY16 9JP United Kingdom; 3 Max Planck Institute for Psycholinguistics, Wundtlaan 1, 6525 XD Nijmegen, The Netherlands; 4 University of Leipzig, Department of Early Child Development and Culture and Leipzig Research Center for Early Child Development, Jahnallee 59, Leipzig, 04109 Germany; 5 Department of Early Prehistory and Quaternary Ecology, University of Tübingen, 72074 Tübingen, Germany

Correction to: *Scientific Reports* 10.1038/s41598-018-30382-0, published online 18 September 2018

This Article contains errors.

In this Article we aimed to put the findings by Smaldino *et al*.^1^ with respect to the sensitivity of the time-window into perspective, by relating them to the exemplified study on social learning in great tits by Aplin and colleagues^2^. Our inference, however, proved incorrect, due to a different interpretation of the ‘events’ in the ‘2,000 threshold’ they identified as producing the sigmoid curve in absence of conformity. We interpreted it as the cumulative sum of events for all individuals, so that 367 birds observing a mean of 6 interactions (as in Aplin *et al*.^2^) would exceed the threshold, whereas the correct interpretation was the number of events for each individual, i.e. 6, thus largely below this threshold.

As a result, the following section of the Discussion is incorrect and should be removed:

“Note also that the time window of ~2,000 observation events that Smaldino *et al*. identify as the threshold after which the sigmoid can be produced in absence of conformity is not that large, given that events in the models represent individual interactions. To put this into perspective, Smaldino *et al*.’s exemplary study in which a time-window (245 seconds) is applied presents evidence for the sigmoid based on more than 2,000 interactions (i.e., 367 birds observing a mean of 6 interactions, leading to a total number of 367*6 = 2,202 observation events). This means that the sigmoid as reported in Aplin *et al*. could have emerged in the absence of individual-level conformity.”

Additionally, this Article contains errors in Reference 1 which was incorrectly given as:

Smaldino, P. E., Aplin, L. M. & Farine, D. R. Do Sigmoidal Acquisition Curves Indicate Conformity? *Sci. Rep*. **8**, 14015 (2018).

The correct reference is listed below as Reference 1.

The Article also contains error in Reference 4 which was incorrectly given as:

Aplin, L. M. *et al*. Experimentally induced innovations lead to persistent culture via conformity in wild birds. **518**, 538–541 (2015).

The correct reference is listed below as Reference 2.

The conclusions of the Article are unaffected by these changes.
